# Invasive Mold Infections in Solid Organ Transplant Recipients

**DOI:** 10.1155/2014/821969

**Published:** 2014-11-23

**Authors:** Yoann Crabol, Olivier Lortholary

**Affiliations:** ^1^Université Paris Descartes, Sorbonne Paris Cité, Centre d'Infectiologie Necker Pasteur, Institut Imagine, Hôpital Universitaire Necker-Enfants Malades, APHP, 75015 Paris, France; ^2^Institut Pasteur, Unité de Mycologie Moléculaire, Centre National de Référence Mycoses Invasives et Antifongiques, CNRS URA3012, 75015 Paris, France

## Abstract

Invasive mold infections represent an increasing source of morbidity and mortality in solid organ transplant recipients. Whereas there is a large literature regarding invasive molds infections in hematopoietic stem cell transplants, data in solid organ transplants are scarcer. In this comprehensive review, we focused on invasive mold infection in the specific population of solid organ transplant. We highlighted epidemiology and specific risk factors for these infections and we assessed the main clinical and imaging findings by fungi and by type of solid organ transplant. Finally, we attempted to summarize the diagnostic strategy for detection of these fungi and tried to give an overview of the current prophylaxis treatments and outcomes of these infections in solid organ transplant recipients.

## 1. Introduction

Solid organ transplantation (SOT) is effective life-sparing modalities for thousands of patients worldwide with organ failure syndromes.

Despite important advances in surgical techniques and immunosuppressive regimens, there remain substantial risks for posttransplantation infections. Because of improvement in diagnosis and treatment of other infections, as Cytomegalovirus infections, invasive fungal infections (IFIs) have now become the leading cause of infection-related mortality following transplantation.

Although SOT populations are at high risk for IFI, with overall incidence rate of 0.9 to 13.2%, respectively [1, 2], they differ with regard to specific defects in host defense mechanisms. Whereas all SOT recipients have dysfunctional T cells and phagocytes, as a result of immunosuppressive drug therapy, disrupted anatomical barriers and iron overload seem to be specific factors favoring fungal infections in lung and liver transplant recipients, respectively.

Those specific defects might explain differences in type, onset, and outcome of IMIs among those populations as reported in two large multicenter prospective studies in the United States and Canada, the Transplant-Associated Infection Surveillance Network (TRANSNET) and the Prospective Antifungal Therapy Alliance (PATH Alliance) studies. Basically, while yeast is major pathogens among SOT recipients (*Candida* sp. and* Cryptococcus* sp. 53% and 8% of IFIs, resp.) [1–3] molds are more prevalent among heart or lung transplants recipients (65% of IFIs). Though rare, endemic fungi (mainly histoplasmosis) represent up to 5.3% of IFIs in endemic areas among SOT recipients [4]. Moreover, median date of diagnosis of IMIs is shorter in liver transplant recipients (99.5 day), compared with 504 days and 382 days in lung and heart transplant recipients.

Among IFIs, invasive mold infections (IMIs) carry the worst outcome [1, 2] and represent an increasing source of morbidity and mortality among SOT recipients [5]. 12-week mortality after the diagnosis of IMIs is the highest among liver transplant recipients (47.1%), compared to kidney, heart, and lung recipients (27.8%, 16.7%, and 9.5%, resp.) [6].

We reviewed specific epidemiology, clinical and imaging findings, diagnostic procedures, treatment, and outcome of proven/probable IMIs, as defined by the 2008 EORTC/MSG criteria [7], in SOT recipient.

## 2. Molds Classification

Molds are filamentous fungi that thrive in soil and decomposing vegetation. Usual molds classification relies on the phenotype of hyphae. Septate hyaline hyphae encompass* Aspergillus* sp. and other Hyalohyphomycosis whereas Mucormycosis, previously termed zygomycosis, belongs to the nonseptate hyaline hyphae. Finally, dematiaceous fungi have melanin-like pigments in the cell walls. They are agents of the phaeohyphomycosis (phaeo is Greek for “dark”). The dematiaceous fungi appear to be especially common in tropical and subtropical regions. Most patients infected with* Rhinocladiella mackenziei* have been reported from Middle Eastern countries, including Saudi Arabia, Syria, or Kuwait [8].

## 3. Epidemiology of Invasive Molds Infections among Solid Organ Transplants

### 3.1. Epidemiology

The epidemiology of IMIs in transplant recipients differs based on geography, host variables, preventive strategies, and methods of diagnosis (see Tables 1 and 2).

Of the 1,208 cases of proven or probable IFI in SOT recipients in TRANSNET, 45 cases of Mucorales,* Fusarium* spp., or* Scedosporium* spp. infection were detected, making these molds the most frequently identified molds after* Aspergillus* (227 cases) within this patient population. The Mucorales (28 patients, 62.2%) were the most common of these molds, followed by* Scedosporium* spp. (11 patients, 24.4%) and* Fusarium* spp. (6 patients, 13.3%). In 10 years of single-center experience recent report, the overall incidence for IMIs among lung, kidney, liver, and heart transplant recipients was 49, 2, 11, and 10 per 1000 person-years, respectively [6]. Among SOT recipients, 17 (37.8%) infections occurred within the first 6 months and 15 (33.3%) occurred >2 years after transplant [2].

Moreover, breakthrough invasive mold infections are an emerging issue among transplant recipients and have been described with the prophylactic or curative use of voriconazole [9], posaconazole [10], caspofungin [11], or polyene [12] antifungal agents. Beside increased minimum inhibitory concentration that remains rare, mechanisms of breakthrough encompass low antifungal serum trough because of noncompliance, insufficient absorption or drug-drug interaction, and low local antifungal concentration because of biofilm or insufficient tissue penetration to critical body site [13].

### 3.2. Invasive Aspergillosis

The proportion of SOT patients within cohorts of patients with invasive aspergillosis (IA) ranges from 8.7% to 44% [14–16]. Overall cumulative incidence of IA ranges from 0.1 to 3.5% in American or European series of adult and paediatric SOT recipients depending on the type of transplant [2, 15, 17–20]. Indeed, among heart, lungs, liver, and kidney SOT patients, the IA incidence was 4.8% (7/146), 4.1% (7/172), 0.8% (9/1067), and 0.3% (11/3157), respectively. The 12-month cumulative incidence of aspergillosis is 2.4%, 0.8%, 0.3%, and 0.1% after lung, heart, liver, and kidney, respectively. In a five-year surveillance of IA in a university hospital, a significant decrease (from 24 to 4 per 100,000 patient days) of IA during the investigation period (2003–2007) was noticed after implementation of a standardized antimycotic prophylaxis protocol [16].

### 3.3. Mucormycosis

The proportion of SOT patients within recent cohorts of patients with mucormycosis varied from 3% to 10% [21–23]. Recently published estimated incidence of mucormycosis in SOT recipients has ranged from 0.4% to 16.0%, depending on the procedure and the geographical area [5, 22, 24]. The TRANSNET study found that the 12-month cumulative incidence of mucormycosis was 0.07% in SOT recipients, with mucormycosis accounting for 2% of invasive fungal infections [25], which is concordant with other studies [2, 3, 26]. Postmortem prevalence evaluation shows that mucormycosis is 10–50-fold less frequent than candidiasis or aspergillosis with a frequency of  1–5 cases per 10 000 autopsies [27, 28]. However, world distribution of mucorales may vary depending on countries. Of note, a study from Iran showed that mucormycosis was the most frequent invasive fungal infection in patients receiving SOT [29]. In the past 2 decades, mucormycosis has emerged as an important invasive fungal infection in SOT and HSCT recipients [21, 30, 31] and an increasing incidence from 0.7/million in 1997 to 1.2/million in 2006 was reported in a population-based study in France [5].

### 3.4. Other Molds

Data regarding epidemiology of hyalohyphomycosis and phaeohyphomycosis are scarcer. Among 162 S. prolificans infections cases retrieved from literature, 8.6% had received solid organ transplantation [32]. Among 73 patients with invasive fusariosis identified through the voriconazole clinical database and the French National Reference Center for Mycoses and Antifungals database, only one was a SOT recipient (kidney) [33].

12-month cumulative scedosporiosis and fusariosis incidence among SOT were 0.024 and 0.012%, respectively, in the TRANSNET study [25]. In a retrospective analysis of 101 cases of primary central nervous system phaeohyphomycosis, 15% had received a solid organ transplant [34]. A 0.7% overall incidence of phaeohyphomycosis among SOT has been recently reported in a retrospective single-center study in New Orleans, USA [8].

### 3.5. Risk Factors

#### 3.5.1. General Risk Factors

Environmental and immune system related risk factors for IMIs have already largely been reviewed by Pagano et al. [35] and Shoham [36].

Moulds are ubiquitous saprophytes present in air, soil, and water that might peak after seasonal periods of dry weather with high temperature [37]. The primary mode of acquiring IMIs is inhalation of fungal airborne spores. The role of the air conidia load in the development of nosocomial invasive aspergillosis, especially during construction and renovation work, has already been demonstrated [38]. In a prospective study, Brenier-Pinchart et al. showed that the outdoor fungal load preceding IA occurrence was significantly higher than that measured during IA-free periods [39].

However, direct inoculation through skin or soft tissue and donor-derived infection have also been reported. In the majority of environment-related cases, IMIs constitute a nosocomial infection, but smoking and living in countryside have been reported as risk factors of IA. If development of infections with resistant emerging mold is related to antifungal selection pressure is still controversial and use of voriconazole has been associated with increased risk for development of mucormycosis in some studies [40–42]. In a case control study of 20 episodes of proven or probable IFI among 744 SOT recipients, prior antibiotic use was more frequent in cases when compared with control subjects and was the only independent risk factor for developing IFI [43], although no clear explanation can be retained currently.

Neutropenia and impaired cell-mediated immunity caused by potent T cell-depleting drugs and antibodies used for immunosuppression in transplant recipients are the most prominent defects in the immune system that predispose individuals to IMIs [22]. In the TRANSNET study, more than 50% patients experienced organ rejection in the 30 days before non-*Aspergillus* mold infection occurred [25]. In a case series of 10 SOT-associated mucormycosis and a review of 106 other cases, 40% of the reported patients had increased immunosuppression to prevent rejection within 1 month before the onset of mucormycosis [44]. However, in other studies, corticosteroid use alone was not specifically identified as a risk factor for mucormycosis [24].

Recent studies have demonstrated the possibility of genetic predispositions to fungal specific immune defect and to the onset of IFIs in case of interleukin-10 excessive production [45], Toll-like receptor [46], long pentraxin 3 (PTX3) [47], plasminogen, or mannose-binding lectin gene polymorphism [48, 49].

#### 3.5.2. Specific Aspergillosis Risk Factors

In a large series of 156 cases of IA in SOT recipients, Gavalda et al. described early and late IA risk factors. The use of vascular amines, hemodialysis, more than one episode of bacterial infection, and cytomegalovirus infection were classified as risk factors for early IA after renal transplantation [19]. Age over 50 years, relapsing bacterial infections, a relapsed malignancy, and chronic graft rejection represent the most prominent risk factors for late IA. Specific organ transplant risk factors for IA are fulminant hepatitis, single lung transplant, early air way ischemia, or pre/posttransplant* Aspergillus* colonization [50].

#### 3.5.3. Specific Mucormycosis Risk Factors

A prospective, matched case-control study in SOT recipients showed that renal failure, diabetes mellitus with or without diabetic ketoacidosis, and prior voriconazole and/or caspofungin use were associated with a higher risk of mucormycosis [24]. Of interest, in this study the use of the calcineurin inhibitor tacrolimus was associated with a 4-fold reduction in the risk of mucormycosis. Finally, iron is a critical growth and virulence factor for Mucorales and high levels of free iron may enhance mucosal damage and may impair cellular antimicrobial systems. Thus, although the direct proof is lacking, liver transplant recipients with iron overload are predisposed to early onset opportunistic infections, including mucormycosis [51].

### 3.6. Health Care Associated IMIs

In a recent review, up to 24% of 169 patients with healthcare-associated mucormycosis were SOT recipients [52]. In this population, its potential nosocomial acquisition with the allograft as the source of early infection has been documented [53]. Graft, especially in the Middle East and Asia, was directly responsible for mucormycosis in 60% of SOT recipients. Primary hepatic mucormycosis occurred only in liver transplant recipients. Major secondary localizations were lungs and gastrointestinal tract. Renal mucormycosis presented as acute rejection in 4 kidney transplant recipients. All of the three patients with a graft artery mucormycosis died.

In a review of 19 cases of invasive fungal infections following commercial kidney transplantation, infecting organisms were* Aspergillus* species (63%), Mucorales(26%), and other fungi (5%). Invasive mold infections were present at the transplanted graft in 35% patients. 76% of patients experienced graft loss or death and 59% died [54].

## 4. Specific Conditions

The type of transplant procedure highly impacts incidence, median time to IFI, and clinical presentation.

### 4.1. Lung Transplant

IMIs are a major source of morbidity and mortality among lung transplant recipients [3, 25, 55–57]. Beyond immune suppression and disrupted anatomical barriers, specific factors favoring fungal infections in lung transplant recipients include ongoing exposure of the graft to environmental fungi, underlying chronic respiratory disease, concomitant sinus, and airway abnormalities impeding mucociliary clearance and blunted cough reflex.

IFIs epidemiology in lung transplant is close to that of HSCT recipients. Indeed, in the TRANSNET study, candidiasis only represented 23% of IFIs in this transplant population whereas IMIs accounted for 70% of all IFIs. Among IMIs, aspergillosis was the most frequent IFI (44% of IFIs), followed by non-*Aspergillus* molds infections (19.8%) and mucormycosis (3%). In the PATH Alliance registry, lung transplanted patients were the only recipients where* Fusarium* and* Scedosporium* infections were encountered [3].

In the TRANSNET study, 12% of lung transplant recipients developed IMI infections [58]. In a retrospective single-center study over 10 years, the overall incidence for IMIs among lung transplants was 49 per 1000 person-years compared to less than 10 per 1000 person-years for other organ transplant recipients [6]. According to the TRANSNET analysis, the 12-month cumulative incidence of IFIs and IMIs in lung transplants recipients was 8.6% and 5 .5%. Patients receiving lung transplants had the highest 12-month cumulative incidence of aspergillosis (4.13%), mucormycosis (0.18%), scedosporiosis (0.2%), fusariosis (0.08%), and the second highest overall incidence of phaeohyphomycosis (1.4%) behind kidney transplant pancreas.

Late-onset IMIs are common in lung transplant recipients. In the TRANSNET subanalysis 52% occurred within 1 year after transplant (median 11 months, range 0–162 months) [58].

Finally, lung transplant recipients are at high risk for the development of invasive aspergillosis at the site of anastomosis between the recipient and the donor trachea [59].

### 4.2. Liver Transplant Recipients

Liver transplant recipients with iron overload are predisposed to early onset opportunistic infections, including mucormycosis. Indeed, liver transplantation has been associated with a 5-fold higher risk of disseminated mucormycosis and shares the highest incidence of mucormycosis with lung transplant (0.16% and 0.18%, resp.). Median time to IFI for liver transplant recipients was significantly shorter than for nonliver SOT recipients (81 days, compared with 533 days, resp.) [3, 25].

## 5. Clinical and Imaging Findings

Clinical manifestations range from colonization or chronic localized lesions to acute invasive and/or disseminated diseases. Whereas no clinical or imaging features of IMIs among SOT are specific for a particular mold, subtle differences exist according to molds species, presence of neutropenia, time of IMIs, and type of organ transplant (see Tables 1 and 2).

Lung involvement seems to be rare in phaeohyphomycosis (7%) compared to other molds where it is the most frequent finding. Tracheobronchial aspergillosis is a particular concern in lung transplant recipients and presents with tracheobronchial ulceration, nodule, pseudomembrane, plaque, or eschar seen on bronchoscopic examination, whereas imaging may not identify the infection [60].

Sinusitis, defined by acute localized pain (sometimes radiating to the eye) or nasal ulcer with black eschar and abnormal imaging findings, including extension across bony barriers is more frequent in mucormycosis (up to 31%) and fusariosis (13–18%) compared to aspergillosis or* Scedosporium* infection (less than 5%). Skin lesions whether primary or metastatic are the most frequent findings in phaeohyphomycosis (up to 89%), fusariosis (up to 70% including onychomycosis), and health care related mucormycosis (up to 57%). Conversely, skin lesions are rare (less than 5%) in aspergillosis compared to scedosporiosis and classic mucormycosis (13–15 and 21%, resp.). CNS involvement is mainly a concern in* Scedosporium apiospermum* infections (50% of cases) and rhinocerebral mucormycosis (14%), especially in cases of* Rhizopus arrhizus* (*oryzae*) infection [21]. Gastrointestinal infection, presenting as severe gastrointestinal bleeding after multivisceral transplantation, has mainly been reported with mucormycosis [61], especially when health care related (up to 12%). Primary hepatic or renal mucormycosis has only been reported in healthcare associated diseases. Disseminated disease occurred especially among scedosporiosis and fusariosis (35 and 35%, resp.) compared to other fungi. Of note, scedosporiosis is associated with a specific risk of intravascular infection in up to 30% of cases.

In a previous case series of 6 fusariosis in SOT recipients,* Fusarium* infections tended to be localized occurred later in the posttransplantation period and had a better outcome compared to patients with hematologic malignancies or bone marrow transplants [62]. We performed a comprehensive review of the literature and identified 20 cases of fusariosis (*Fusarium* sp., *n* = 9;* F. solani complex*, *n* = 7;* F. proliferatum*, *n* = 2;* F. oxysporum* and* F. verticillioides*, *n* = 1 each) among SOT recipients (M/F sex ratio = 1; median age 48 years [18–66]), including lung (*n* = 10) kidney (*n* = 6), liver (*n* = 3), and heart-liver transplant (*n* = 1). Median time to fusariosis was 365 days after SOT [5–5475] and tended to be earlier in cases of disseminated disease (122 days [11–1460]). Pneumonia (*n* = 9) and skin infections (*n* = 8) were more common among lung transplants and nonlung transplants, respectively. None of the kidney transplant recipients presented disseminated fusariosis that occurred in 25% of cases among lung and liver transplants recipients. Health care or preventable infections occurred in 3 patients (venous or peritoneal catheter infections, *n* = 2, and onychomycosis, *n* = 1).

In a retrospective single-center study among 479 consecutive heart transplant recipients, late aspergillosis (occurring >3 months after transplantation) showed a higher frequency of disseminated disease and involvement of the central nervous system or atypical sites compared with early (first 3 months) episodes [20].

Park et al. compared the clinical features and radiologic findings of 27 SOT with those of 35 neutropenic patients with invasive pulmonary aspergillosis (IPA). The SOT recipients were less likely to have fever (22%) and more likely to present with intercurrent bacterial pneumonia (67%) or require mechanical ventilation (89%). Most of them (≈30%) presented with macronodules, mass like consolidation, or “airway invasive” pattern as peribronchial consolidation or ground-glass opacity on chest CT scan. They were less likely to have other “angioinvasive patterns” as halo signs (8%) or air-crescent signs (0%), classically described in neutropenic patients [63].

## 6. Diagnostic Strategy

In high risk patient cohorts, such as patients after solid organ transplantation, early diagnosis of IFIs is essential, as delayed or missing diagnosis of IFI results in increasing rates of mortality [64]. However, diagnosis of most IFIs, especially IMIs, is difficult because conventional assays, that is, direct examination and culture, have low sensitivity and specificity, and radiology often provides nonspecific and transient results. In addition, the contribution of no-conventional tests is lower than that observed among neutropenic patients (Table 3). Of note, EORTC diagnostic criteria have been developed in order to maintain consistency in clinical and epidemiologic studies, not to drive therapeutic decision making.

### 6.1. Conventional Assays

#### 6.1.1. Microscopy Techniques

Microscopy techniques include fresh and stained examination of microbiological samples, as well as histopathological studies. Optical brighteners (calcofluor or blankophor) with 10 percent potassium hydroxide or Grocott-Gomorri methenamine silver stain, haematoxylin and eosin, and periodic acid Schiff stain can be used to stain respiratory specimens, cytology, or histopathology preparations. Whereas special Fontana-Masson stain is the most sensitive for melanin and phaeohyphomycosis detection, silver stains obscure fungi so that the brown color of phaeohyphomycosis cannot be seen. Microscopy techniques are useful to distinguish hyaline molds (septate hyalohyphomycetes and nonseptate Mucorales) from pigmented phaeohyphomycetes. However, those methods have important limitations, one being their low sensitivity, especially if systemic antifungal therapy has already been started. Furthermore, all hyaline hyphomycetes, including* Aspergillus*,* Scedosporium*,* Fusarium*,* Acremonium*, and* Paecilomyces* exhibit similar appearance in clinical specimens under microscopic examination and correlation with culture results is needed for definitive identification [65]. It is important to point out that, unfortunately, the samples taken are sometimes sent for histopathological and cytological evaluation only and not for microbiological study [66].

#### 6.1.2. Culture

Although most fungi grow on standard culture media, such as blood agar and chocolate agar, media specific for fungal growth must also be used, including malt extract agar, cornmeal agar, Sabouraud glucose agar with cycloheximide, potato agar, and brain heart infusion agar. Selective media supplemented with cycloheximide or benomyl allow growth of* Scedosporium* over other filamentous fungi from bronchial secretions. Cultures must be incubated at 30 and 37°C.

Cultures are usually positive after 1 to 3 days of incubation except for slow-sporulating species (e.g.,* Aspergillus lentulus*,* Neosartorya udagawae*). Once isolated in culture, molds are primarily identified based on their macroscopic morphology on culture media and type of reproductive structures observed microscopically. If possible, EORTC/MSG recommends appending the identification at the genus or species level from the culture results. In the TRANSNET study, 24 out of 358 proven or probable IMIs among SOT recipients (6.7%) were culture negative. Previous systemic antifungal therapy and aggressive processing of the specimens before plating are associated with false negative cultures. Grinding of specimens should therefore be avoided especially for Mucorales. Conversely, culture isolation of molds species from the airways does not necessarily indicate invasive disease. Indeed, respiratory tracts of up to 40% of lung transplant recipients become colonized with* Aspergillus *spp. [67]. Importantly, recovery of* Aspergillus* spp. from an airway sample in lung transplant recipients warrants a bronchoscopic examination to exclude the presence of tracheobronchitis because radiographic and imaging studies may be nonrevealing at this stage. Blood culture yield is usually very low except for* Fusarium* and* Scedosporium* sp. Recovery of* Aspergillus* species from blood cultures invariably represents contamination.

Of the 116 SOT recipients with mucormycosis reported in a literature analysis, 61 (52.6%) received a diagnosis on the basis of both biopsy and culture, 45 (38.8%) on the basis of biopsy only, 7 (6%) on the basis of a positive culture result with a compatible clinical presentation, and 3 (2.6%) on the basis of either culture or biopsy [44]. In another older study among patients with hematological malignancies, only 25% of sputum or bronchoalveolar lavage (BAL) were positive for Mucorales [68]. Although contamination of clinical specimens by Mucorales is possible, positive culture results, especially when repetitive and associated with positive smear results, strongly suggest mucormycosis after transplantation.

### 6.2. New Assays

Because of the limitations of conventional methods, alternatives to culture techniques have been developed to try to diagnose IFIs earlier, by detection of fungal cell components.

Recent assays include antigen detection systems, such as galactomannan or 1,3-*β*-d-glucan, mass spectrometry, and different molecular methods (PCR assays), which allow faster diagnoses with increased sensitivity and specificity.

Galactomannan (GM) is a polysaccharide that is a major constituent of* Aspergillus* cell walls and that is also present in the cell walls of a number of other fungal species, except Mucorales. Platelia Aspergillus enzyme immunoassay for GM antigen detection in serum is an important tool for both diagnosis and outcome evaluation of invasive aspergillosis. In a meta-analysis Pfeiffer et al. assessed the accuracy of a GM serum assay for diagnosing invasive aspergillosis. While the specificity of the test was good (84%), the sensitivity of the test among SOT was significantly lower compared to bone marrow transplant (22% and 82%, resp.). Of note, further study assessed the utility of GM antigen detection in BAL for the diagnosis of IA among 116 lung transplants. Increasing the index cutoff value to > or =1.0 yielded a sensitivity of 60%, a specificity of 98%, and positive and negative likelihood ratios of 28 and 0.40, respectively [69]. In a recent Brazilian study, Nucci et al. reviewed the records of 18 patients with invasive fusariosis and GM evaluation. The sensitivity and specificity of GM were 83% and 67%, respectively. GM was positive before the diagnosis of invasive fusariosis in 73% of the cases at a median of 10 days [70]. In patients with a possible invasive fungal infection, negative galactomannan test results in serum and BAL increase the likelihood of invasive mucormycosis [71, 72]. Conversely, a positive GM or 1,3-*β*-d-glucan result does not exclude the diagnosis of mucormycosis, because of the cooccurrence of fungal infections (up to 45% in a single-center study) [42].

The 1,3-*β*-d-glucan (BG), a cell wall component of most fungal species, can be detected in blood during IFD. The BG test is used as a diagnostic tool for the detection of a broad spectrum of fungal pathogens, with the exception of Mucorales and* C. neoformans*. This test is currently used in combination with classical clinical, radiological, and microbiological findings. It was included in the updated consensus definition of probable IFD by the EORTC/MSG consensus [7]. In 2012, a meta-analysis reported the performance of BG antigenemia assays for the diagnosis of IFD in patients with hematological malignancies, including allogeneic HSCT [73]. This paper highlighted the fact that two consecutive positive BG tests strongly suggested the diagnosis of probable IFD according to a very high specificity (99%) and positive predictive value. Thus, following two positive BG tests, a preemptive antifungal therapy should be started. However, despite the excellent specificity of the assay, the sensitivity of BG remains low, and a negative result could not rule out the diagnosis of fungal disease.

Matrix-assisted laser desorption ionization time-of-flight (MALDI-TOF) mass spectrometry provides protein spectra from crude extracts or intact cells. Each bacterial or fungal species can be identified within minutes by comparison of its own characteristic spectrum with that in a reference spectra library, at least for common fungal species. Fungi such as Aspergillus,* Fusarium* [74],* Lichtheimia* species [75], or* Scedosporium* [76] were thus identified with MALDI-TOF mass spectrometry.

A range of different PCR assays (conventional, nested, and real-time) has been developed, targeting different gene regions (mainly 18S, 28S, internal transcribed spacer, and D1/D2 of the rRNA or *β*-tubulin), including a variety of amplicon detection methods. These molecular assays provide high potential in terms of sensitivity and specificity but vary widely in their feasibility. According to recent recommendations from European Society of Clinical Microbiology and Infectious Diseases (ESCMID) and European Confederation of Medical Mycology (ECMM), regarding diagnosis of IMIs, molecular methods are recommended for accurate identification of mucormycosis and phaeohyphomycosis, especially in case of outbreak and to a less extent for hyalohyphomycosis identification. In the TRANSNET study, over 10% of the isolates associated with IA in transplant recipients were found to be cryptic species and molecular identification methods were essential in distinguishing them [77].

Level of evidence for fungal detection by use of PCR methods is still lower and not supported by current recommendations. Harmonization of PCR-based techniques is necessary before any clear recommendations can be made regarding their clinical utility. However, results are promising, especially for* Aspergillus* and Mucorales. Luong et al. compared the performance of publicly available pan-Aspergillus specific real-time polymerase chain reaction (PCR) assays with the Platelia galactomannan (GM) assay in 150 bronchoalveolar lavage (BAL) samples from lung transplant recipients. The sensitivity and specificity of pan-Aspergillus PCR and GM (>0.5) for diagnosing IPA were 100% and 88% and 93% and 89%, respectively. Positive results for both BAL PCR and GM testing improved the specificity to 97% with minimal detriment to sensitivity (93%) [78]. An in-house seminested PCR that targets the 18S ribosomal DNA of Mucorales was evaluated on fresh tissue specimens obtained from 46 patients with a haematological malignancy and 15 patients with solid-organ transplantation. Seminested PCR detected Mucorales in all specimens with nonseptate hyphae (*n* = 13, sensitivity 100%) whereas culture remained negative in five cases [79]. Both formalin-fixed paraffin-embedded and fresh or frozen tissue samples can be used, with a lower yield using the former [80].

Mucorales-specific T cells were investigated in 28 hematologic patients during the course of their treatment. Mucorales-specific T cells could be detected only in patients with Mucormycosis, both at diagnosis and throughout the entire course of the disease, but neither before nor for long after resolution of the infection. This assay might be a promising surrogate diagnostic marker of mucormycosis [81].

Positron emission tomography using 18 F-fluorodeoxyglucose (18 F-FDG PET) has proven useful for more accurate evaluation of IFD extent [82]. It can detect small infectious foci before the onset of the anatomical abnormalities assessed by conventional radiological tools but is limited by the size of the lesions (<7-8 mm). Some other limitations are the impossible distinction between infection and inflammation, the physiologic uptake of 18 F-FDG in the heart, brain, and kidney, and the dose rate identical to that for a conventional computed tomography scan. Various mold and yeast infections have been associated with a pathological uptake of 18 F-FDG, including Mucorales,* Candida* spp., and* Scedosporium* spp. [82]. Further studies are needed to assess its usefulness in treatment efficacy follow-up in transplantation.

### 6.3. Diagnostic Strategy

As soon as a clinical scenario is consistent with IMIs, blood cultures, BG, CT of the brain, sinuses, and chest (with bronchoscopy, bronchoalveolar lavage, and detection of GM antigen in BAL when scans of the chest show abnormalities) should be performed [71]. Detection of serum galactomannan (GM) should not be used for routine diagnosis of IFIs in SOT recipients. Transthoracic percutaneous needle aspiration or video assisted thoracoscopic biopsy is standard procedures for establishing a diagnosis of IMIs in difficult cases.

A proven diagnosis of IMI can be based on positive blood cultures that yield particular filamentous fungi (*Scedosporium* spp.,* Fusarium* spp.), observation of tissue with invasive fungal structure, or isolation from sterile tissue or fluid. The detection of mould nucleic acid by PCR in BAL or sputum should be considered particular in lung/heart transplants recipients patients due to the subsequent risk of invasive infection.

## 7. Treatment

Without adequate therapy, IMIs will almost always progress to dissemination to the CNS and/or other critical organs and ultimately relentless fatal disease. Because of the potential progression of these infections, empiric antifungal therapy should be initiated early in SOT patients with high suspicion of IMIs while diagnostic evaluation is undertaken.

In lung transplant recipients, colonization with* Aspergillus* sp. must be treated to prevent invasive disease, with nebulized liposomal amphotericin B plus removal of the debris by repeated bronchoscopies.

With the exception of invasive pulmonary aspergillosis, where guidelines rely on randomized controlled trials among patients mainly with hematological diseases [83], no solid recommendations can be provided for antifungal treatments, given the scarcity of data and the potential publication bias.

### 7.1. General Considerations

The cornerstones of treatment of IMIs rely on early systemic antifungal therapy, assessment of the need for surgical debridement, and reversal of immunosuppression.

When amphotericin B is considered, lipid-based preparations should be favored over amphotericin B deoxycholate as they exhibit fewer side-effects especially among SOT recipients [24] and appeared superior to that of deoxycholate amphotericin B for treatments of fusariosis [84] and Mucorales [85].

The optimal duration of treatment of IMIs has not been studied prospectively and is generally unknown. Therapy should be continued throughout the period of immunosuppression and until complete and permanent response demonstrated by imaging, usually with a minimum of 6–12 weeks. For patients with successfully treated IMIs who will require subsequent immunosuppression, resumption of antifungal therapy can prevent recurrent infection.

Of note, azole drug has important drug-drug interaction issues with immunosuppressants commonly used for SOT or anticonvulsant therapy used in case of CNS involvement.

As an example, if voriconazole is administered, the calcineurin inhibitor dose should be reduced by 50–60% [86] and sirolimus discouraged. If the patient receives posaconazole, then the dose of tacrolimus or cyclosporine A should be reduced by 60–75% and 14–29%, respectively [87]. Conversely, few drug-drug interactions affect the echinocandins. Caspofungin presents the highest rate and anidulafungin the lowest [88]. In addition, long-term voriconazole prescription has been associated with fluoride excess, periostitis [89], and phototoxicity involving acute skin lesions followed by actinic keratosis and ultimately squamous cell cancer [90].

Therapeutic drug monitoring is recommended if itraconazole, voriconazole, or posaconazole is prescribed; monitoring is highly recommended in unsatisfactory response to therapy, suspicion of toxicity or drug interactions, impaired liver, or renal function, gastrointestinal disturbances [91] and also in patients on extracorporeal membrane oxygenation. Indeed, therapeutic drug monitoring of voriconazole (in order to maintain the range between 2 and 4 *μ*g/mL) has been shown to reduce drug discontinuation due to adverse events and improve the treatment response in invasive fungal infections [92].

Low posaconazole concentrations have been shown to be common, especially in case of diarrhea or coadministration with proton pump inhibitors or metoclopramide. Low concentrations are associated with breakthrough fungal infection, supporting the utility of monitoring posaconazole concentrations to ensure optimal systemic exposure (1 *μ*g/mL) [93].

### 7.2. Antifungal Therapy

#### 7.2.1. Aspergillosis

For primary treatment of invasive pulmonary aspergillosis, IV or oral voriconazole is recommended for most patients. For seriously ill patients, the parenteral formulation is recommended although voriconazole has high bioavailability [94]. L-AMB may be considered as alternative primary therapy in some patients. Of note, L-AMB (3 mg/kg/day and 10 mg/kg/day) has similar efficacy but greater toxicity at higher dose. For salvage therapy, options include switch or addition of antifungal drugs from different classes using an AMB formulation or an echinocandin (caspofungin or micafungin). Additional use of an azole (posaconazole, itraconazole) should take into account prior therapy and pharmacokinetic considerations. Isavuconazole, a novel broad spectrum triazole agent, has in vitro activity against most medically important fungi. Due to a once-a-day regimen and absence of cyclodextrin nephrotoxic compound, it has the potential to become first line therapy for invasive aspergillosis [95]. Recently presented results of a study comparing isavuconazole and voriconazole showed a similar efficacy of both drugs while safety was better in those treated by isavuconazole [96].

Imaging and GM have been shown useful tools for follow-up. Of importance, the volume of pulmonary infiltrates may increase for the first 7–10 days of therapy, especially in the context of granulocyte recovery. Chai et al. showed that among hematological patients and HSCT transplants, a GM reduction of >35% between baseline and week 1 predicted a probability of a satisfactory clinical response. Conversely, every 0.1-unit increase in the GM between baseline and week 2 increased the likelihood of an unsatisfactory clinical response by 21.6%. However, resolution of GM to a normal level is not sufficient as a sole criterion for discontinuation of antifungal therapy.

#### 7.2.2. *Scedosporium*

In vitro and in vivo data show that* P. apiospermum* is resistant to amphotericin B and flucytosine and demonstrates variable susceptibility to itraconazole, voriconazole, posaconazole, and micafungin.* S. prolificans* is resistant to caspofungin, polyenes, and azoles. Voriconazole demonstrated the strongest in vitro activity on both of these species [97]. A recent in vitro study suggests synergistic activity of the antibacterial agent colistin with voriconazole against* Scedosporium* spp. [98]. Success of combinations therapy of azole and terbinafine has been reported in small case series [99, 100].

#### 7.2.3. Fusariosis

In immunocompromised patients, voriconazole and lipid-based amphotericin B formulations have been reported with success [33, 101] and are supported as first line therapy by ESCMID and ECMM. Posaconazole is recommended as salvage therapy [102]. Data on combination therapy for fusariosis are limited to a few case reports.

#### 7.2.4. Mucormycosis

Immediate treatment initiation in case of mucormycosis is strongly supported to increase survival rates. Indeed, in an uncontrolled study in patients with haematological malignancy the 12-week mortality rate increased twofold with medical treatment deferred for >6 days from onset of symptoms [103]. Liposomal amphotericin B is the drug of choice and the dose should be at least 5 mg/kg/day, as suggested by murine model [104], uncontrolled retrospective study [105], or analysis of registry [23, 30]. Lanternier et al. evaluated the feasibility and efficacy of high dose (10 mg/kg/day) of liposomal amphotericin B for the initial treatment of mucormycosis. Treatment was feasible in more than half of the patients despite frequent (40%) renal toxicity. At week 12 the response rate was 45% [106]. In a retrospective study, combination therapy with an amphotericin B formulation and caspofungin has been described as successful in a limited number of predominantly diabetic patients with rhinocerebral mucormycosis [107]. Preclinical data indicate that the combination of a lipid formulation of amphotericin B and posaconazole is unlikely to be effective [108]. The duration of acute phase treatment is not defined. Minimum treatment duration should be 6–8 weeks or until resolution of all associated symptoms and findings.

Maintenance therapy has to be considered in persistently immunocompromised transplant recipients. After the infection has stabilized, the standard treatment recommendation is oral posaconazole (800 mg/day) [71].

For salvage treatment posaconazole 200 mg four times daily, yielding the highest exposure, is strongly recommended, with response rate ranging from 60 to 80% [109, 110]. New posaconazole formulations, delayed-release tablet and an IV formulation recently approved by the US Food and Drugs Administration, might enhance its effectiveness.

#### 7.2.5. Phaeohyphomycosis

Because of lack of clinical trials and varied resistance patterns among species, there are no standardized therapies for infections caused by phycomycetes. However, voriconazole, posaconazole, itraconazole, and in some cases amphotericin B demonstrate the most consistent in vitro activity against this group of fungi. Voriconazole may have advantages for central nervous system infections because of its ability to achieve good cerebrospinal fluid levels with brain/plasma ratios ≈3.0 at steady state [111], unlike itraconazole. Combination antifungal therapy for cerebral abscesses and for disseminated infections in immunocompromised patients has been suggested [112]. As an example, in a retrospective review of 101 cases of primary central nervous system phaeohyphomycosis, including 15 SOT recipients, treatment with the combination of amphotericin B, 5-FC, and itraconazole was associated with improved survival, compared with other medical regimens, with 17% mortality versus 74% mortality [34].

### 7.3. Surgery

Of major importance, extensive early surgical debridement is generally recommended in combination with antifungals, at least during non-Aspergillus mold infections. Indications for surgery are summarized in Table 3. Whereas no well-designed randomized clinical efficacy trial has been published to date, the highest level of evidence for the need of surgery has been provided in mucormycosis. In this setting, surgery was reported to be an independent predictor of successful therapy and survival in SOT recipients with pulmonary or rhino-orbital-cerebral mucormycosis [105, 113]. In a retrospective study on 30 patients combined with a literature analysis of 225 patients with mucormycosis, surgical debridement of lung involvement was associated with a decrease of mortality from 62% to 11% [114]. In a recent retrospective case series of 22 consecutive rhino-orbital- cerebral mucormycosis adults, none versus 75% died, on day 90 with or without local control, respectively [115]. Indications of surgery in IA depend on multiple variables, severity of lesion, surgical judgment, and the ability of the patient to tolerate the operative procedure, as well as the potential role of alternative medical therapy [60]. For* Scedosporium*, surgical resection remains the key to a successful outcome if the lesions are localized and is sometimes the only available therapy regarding pan-resistance to antifungal treatment. In addition to antifungal treatment, the optimal management of patients with fusariosis includes surgical debridement of infected tissues and/or nails [116] and removal of venous catheters in confirmed catheter-related fusariosis. For cutaneous and CNS phaeohyphomycosis, complete excision is superior to aspiration and is generally required for the disease to resolve [34].

### 7.4. Improvement of Immune and Metabolic Factors

Paramount to the successful treatment of IMIs in SOT recipients is improvement of immune and metabolic factors, by administering tapering doses of steroids and immunosuppressive agents, when feasible, and by controlling hyperglycemia, especially in the case of mucormycosis [71]. Moreover, neutropenic patients who are not receiving a colony-stimulating factor may benefit from the addition of granulocyte colony-stimulating factor or granulocyte-macrophage colony-stimulating factor [71, 117, 118].

### 7.5. Other Approaches

The role of newer generation iron chelation agents (e.g., deferasirox) is controversial. Indeed, the iron chelator deferasirox protects mice from mucormycosis and enhanced the efficacy of liposomal amphotericin B [119]. In contrast, a recent double-blinded, randomized, placebo-controlled but small size study (*n* = 20) mostly in haematological patients has failed to demonstrate a benefit of combination therapy of L-AMB with deferasirox. Instead, patients with mucormycosis treated with deferasirox had a higher mortality rate at 90 days. Population imbalances in this small study make generalizable conclusions difficult [120]. For these reasons, in haematological patients with mucormycosis, adjunctive treatment with deferasirox is discouraged, whereas in other patient groups it is recommended with marginal strength by ESCMID and ECMM.

Other therapeutic approaches, such as hyperbaric oxygen therapy [121], lovastatin [122], or the use of new antifungal agents active against Mucorales, such as isavuconazole, have not been clinically validated.

## 8. Prophylaxis

Due to the lack of clinical trials and to the epidemiological differences in IFD in different transplant programs, there are no definitive recommendations for the prevention of IFD in SOT. Because of overlapping epidemiology, prevention strategies for invasive candidosis and aspergillosis must be combined in certain transplant populations. According to recent ESCMID Study Group for Infections in Compromised Hosts (ESGICH) recommendations [123], there are the following.

(i) Universal prophylaxis against* Aspergillus* is indicated in lung or lung/heart transplant recipients. The efficacy and advantages of using nebulized lipid formulations of amphotericin B have been demonstrated. Local irritation symptoms occur in fewer than 10% of patients, and the use of salbutamol or halving the drug concentration can improve the symptoms. Alternatively, antifungal prophylaxis can be performed with azoles such as itraconazole or voriconazole. However, long term voriconazole related hepatotoxicity and skin cancer are limiting issues.
(ii) No antifungal prophylaxis is required in kidney transplant recipients.
(iii) In the other settings, prophylaxis against* Aspergillus* should only be discussed in case of specific risk factors, such as acute rejection, poor initial allograft function, hemodialysis, anastomotic problems, need for reintervention, CMV disease, excessive* Aspergillus* spp. in the air of the centre, or overimmunosuppression. Echinocandins, without renal toxicity and few drug-drug interaction favors over L-amphotericin B in high risk liver, pancreas or intestinal transplant recipients. Itraconazole has been shown to be effective among high risk heart transplants.

There is no agreement about the prevention strategy for late IA (>90 days after transplant). In patients with risk factor of late IA as chronic rejection, allograft dysfunction due to HCV (in liver transplant), hemodialysis, overimmunosuppression, and transplant-related neoplasms prophylaxis should be considered.

## 9. Prognosis and Outcomes

While not as high as among patients with neutropenia, twelve-week overall mortality in SOT with IMIs remains significant and ranges from 29.4% to 35.6% (compared to 46% among patients with neutropenia [63]) according to large available PATH, SAIF, or TRANSNET studies [14, 15, 124].

Furthermore, fungal species, disease extension, type of transplant procedure, time to IMIs, underlying organ damage, and treatment highly impacted mortality rate.

The overall mortality rate among SOT recipients with mucormycosis is generally 38%–48% [22, 44]. In the PATH alliance database, mucormycosis was associated with the worst IMIs outcome, with more than 55% mortality rate by 12 weeks. In a review of 20 cases of fusariosis among SOT recipients, 8 (40%) died. Finally, the outcome of* S. prolificans* infection is very poor, because no drug appears to be effective.

In SOT patients, renal or hepatic insufficiency, malnutrition, central nervous system disease, and disseminated disease independently predicted poor outcomes [24, 124]. Moreover, among SOT patients with IA who received antifungal therapy, use of amphotericin B preparation as part of initial therapy was associated with increased risk of death [124]. Interestingly, in heart transplant recipients, there was a trend toward lower related death in early versus late cases of IA (26% versus 63%) [20].

In the TRANSNET study, liver transplant recipients were more likely to die after IA [3]. These data were confirmed in a recent retrospective single-center analysis among 106 patients with IMIs, where the 12-week mortality among liver, kidney, heart, and lung recipients was 52.4%, 47.1%, 27.8%, 16.7%, and 9.5%, respectively [6].

## 10. Conclusion

Although their outcomes have improved in the current era, IMIs remain a significant and severe posttransplant complication in SOT recipients. Epidemiology of IMIs is changing with an increasing rate of late infection and breakthrough of emerging molds species sometimes resistant to antifungal prophylaxis. Since early diagnosis and treatment are critical, there is a need for prospective validation of the new diagnostic tools already available. Preventive strategies as patient education and information regarding environmental risk factor, special attention to health care related infections, and proper handling of immunosuppressive drugs are crucial, while waiting for more evidence based recommendations regarding optimal antifungal prophylaxis.

Patient's tailored surgical and medical antifungal treatments, taking account of transplant procedure and toxicities risks, drug-drug interactions, fungal species identification, and in some cases antifungal susceptibility are of major importance. Follow- up biomarkers or PET-CT seems to be promising tools to guide treatment.

## Figures and Tables

**Table 1:** Epidemiology, clinical and imaging findings among SOT recipients with invasive mold infection.

	Aspergillosis	Scedosporium	Fusariosis	Mucormycosis	Phaeohyphomycosis
*Epidemiology* [2, 8]					
Number (%) among proven or probable IFIs in SOT recipients	227/1208 (18.8%)	11/1208 (0.9%)	6/1208 (0.5%)	28/1208 (2.3%)	NA (<5%)
12 months of cumulative incidence	0.7% Lung 4.13%	0.024% Lung 0.2% kidney 0.01%	0.012% 1 Lung 0.08% 1 Liver 0.02%	0.07% Lung 0.18% Liver 0.16% Early onset (<90 d) for liver Late onset for lung (>90 d)	0.7% overall CI Lung 1.4% Heart 1.2% Kidney 1% Liver 0.16% Kidney/pancreas 3.6%
Median time to IMI, days	184	467	467	312	700

Common species [2, 8, 14, 25, 44]	*A*. *fumigatus* 60–73% *A*. *flavus* 7–10% *A*. *niger* 6–9%	*S. apiospermum* 55% *S. prolificans* 45%	Unspecified 45–50% *F. solani* 17–35% *F. proliferatum* 0–10% *F. oxysporum*0–33%	*Rhizopus* 57–73% *Mucor* (13–25%) *Lichtheimia* (0–4%) *Cunninghamella* (0–14%)	*Exophiala jeanselmei* & sp. 41% *Ochroconis gallopava*11% *Alternaria* species 11% *Phoma* species 11%

Clinical findings [2, 8, 14, 44, 57, 125]	**N** = 227	**N** = 23	**N** = 6**–20**	**N** = 2**8–116 **	**N** = 27
Sinus (%)	4	4	0–18	13–31	rare
Lung (%)	78 (tracheobronchial 5%)	52	39–45	22–56	7
Skin (%)	4	21	45–70 (including onychomycosis)	13–15	89
CNS (%)	2	49	rare	14 (rhinocerebral)	7
Disseminated (%)	10	35 (30% of intravascular infection)	25	9–12% (including gastrointestinal)	4%

Lung CT findings^*^ [40, 63, 126–129]	**In nonneutropenic patient:** *Airway invasive pattern:* Peribronchial consolidation (31%) Ground-glass opacity (38%) *Angioinvasive pattern:* Macronodules (35%) Mass-like consolidation (27%) Halo signs (8%) Air-crescent signs (0%) Reversed halo sign (<1%)	In HM: Multiple bilateral patchy nodular condensations, alveolar infiltrates or, most commonly, consolidation without cavitation	In HM: Pulmonary nodules in 82% Reversed halo sign 0% Halo sign <20%!	in HM: Focal consolidation, masses, pleural effusions, or multiple nodules Reversed halo sign (19%) Among patients with HM, predictors of mucormycosis versus IPA are concomitant sinusitis, >10 pulmonary nodules on CT scan, and pleural effusion	NA

CI: cumulative incidence; HM: patients with hematological malignancies; IMI: invasive mold infection; NA: not available.

^*^No specific data among SOT except for invasive aspergillosis.

**Table 2:** Characteristics of IMIs by type of SOT.

	Liver	Lung	Kidney	Heart
Species distribution (% among IFIs) [3]	Candidiasis 78.7% *Aspergillus sp*. 7.9% *Crypto sp.* 7.1% Zygomycetes 3.1% Other molds 0%	Candidiasis 23.9% *Aspergillus sp.* 63% *Crypto sp.* 2.2% Zygomycetes 2.2% Other molds 9.7%	Candidiasis 60.6% *Aspergillus sp.* 11.9% *Crypto sp.* 19.3% Zygomycetes 0.9% Other molds 2.7%	Candidiasis 65% *Aspergillus sp*. 25% *Crypto sp.* 2.5% Zygomycetes 2.5% Other molds 2.5%

Median time to IA, days [3]	99.5	504	—	382

Risk factors for IA [50]	Re-transplantation* * Renal failure, particularly hemodialysis transplantation for fulminant hepatic failure* * Reoperation	Single lung transplant* * Early airway ischemia* * CMV infection Rejection and augmented immunosuppression Pretransplant *Aspergillus* colonization* * Posttransplant *Aspergillus* colonization within a year of transplant* * Acquired hypogammaglobulinemia	Graft failure requiring hemodialysis* * High and prolonged duration of corticosteroids	Isolation of *Aspergillus* species in respiratory tract cultures Reoperation* * CMV disease* * Posttransplant hemodialysis* * Existence of an episode of invasive aspergillosis in the program 2 months before or after heart transplant

Site of IMIs	Lung 70.6% Sinus 17.6% Skin and soft tissue 35.3%	Lung 100% Sinus 9.5% Skin and soft tissue 0%	Lung 85.3% Sinus 5.6% Skin and soft tissue 11.1%	Lung 66.7% Sinus 16.7% Skin and soft tissue 33.3%

Chest CT findings for patients with IA	Nodular lesions 0% Infiltrates/consolidations 73%	Nodular lesions 20% Infiltrates/consolidations 80%	Nodular lesions 50% Infiltrates/consolidations 50%	Nodular lesions 67% Infiltrates/consolidations 33%

IA: invasive aspergillosis, CMV:*Cytomegalovirus*, IFIs: invasive fungal infections, and IMIs: invasive molds infections.

**Table 3:** Diagnosis of invasive mold infection among solid organ transplant recipients.

	Aspergillosis	Scedosporium	Fusariosis	Mucormycosis	Phaeohyphomycosis
Pathogen detection					
Microscopy techniques [130]					
Color	Hyaline	Hyaline	Hyaline	Hyaline	Brown
Size	3–8 microns wide	3–8 microns wide	3–8 microns wide	5–15 microns wide	Variable
Septation	Yes (no adventitious forms)	Yes (+/−adventitious forms: yeast-like structures)	Yes (+/−adventitious forms: yeast-like structures)	No or pseudoseptation	Yes
Branching	Dichotomous acute angle (45°)	Dichotomous acute angle (45°)	Dichotomous acute/right angle	Irregularly right angle	Variable

Culture [3, 57, 125]	*Among 128 IA in SOT: *All samples: Sens: 91.4% Sputum or BAL: PVP: 58%^**^ Blood culture: limited utility	*Among 23 SOT*: No positive blood culture *In HSCT:* Blood culture Sens: 50%	*Among* *SOT* + *HSCT*: blood culture Sens: 41%–60%	Blood culture: limited utility Culture of CSF and urine often negative for kidney or CNS infection	Blood culture: limited utility

PCR [72, 126, 131, 132]	*Among HSCT:* Blood: Sens 0.88, Spe 0.75 (0.87 if 2 consecutive positive samples). High NPV *Among lung SOT: * BAL: Sens 100%, Spe 88% (pan-Aspergillus PCR)	Insufficient data	Insufficient data	Moderately supported. Fresh material is preferred over paraffin-embedded tissue because formalin damages DNA.	Insufficient data

Beta-D-glucan assay [133, 134]	Among SOT: Sens: 66% Spe: 44%	NA	Few data but case report with positive results	Negative	Few data but case report with positive results

Galactomannan Ag [70, 135, 136]	*Among SOT* Serum/BAL: Sens: 22%/81.8% Spe: 84%/95.8%	NA	*In HSCT: *Sens 83% & Spe 67% Positive before the diagnosis in 73% at a median of 10 days	Negative	Cross reactivity in some cases Not recommended

Identification species by molecular method [72, 126, 132, 137]	Required in ≈10% of cases because of cryptic species with particular antifungal resistance pattern	Marginally recommended	Marginally recommended	Recommended to establish epidemiological knowledge (and in case of healthcare-associated mucormycosis and outbreaks)	Recommended especially for unusual or newly described pathogens.

Antifungal susceptibility testing to guide treatment [72, 126, 132]	Not recommended in routine in area of low frequency of resistance.	Marginally recommended	Marginally recommended	Moderately recommended	Strongly recommended for deep infections

Ag: antigen; AMB: amphotericin B; BAL: bronchoalveolar lavage; FC: flucytosine; ITC: itraconazole; HM: patients with hematological malignancies; IMI: invasive mold infection; NA: not available; NVP: negative predictive value; PCZ: posaconazole; PVP: predictive positive value; Sens: sensitivity; Spe: specificity; SOT: solid organ transplant recipients; VCZ: voriconazole; no specific data among SOT except for invasive aspergillosis; ^**^lower PVP in lung transplant recipients.
